# Surgery Follows Function: Redo Mitral Valve Replacement After Cardioband Implantation

**DOI:** 10.1016/j.atssr.2022.12.010

**Published:** 2022-12-24

**Authors:** Anja Osswald, Arjang Ruhparwar, Bastian Schmack

**Affiliations:** 1 Department of Thoracic and Cardiovascular Surgery, University Hospital Essen, Essen, Germany

**Figure 1:**
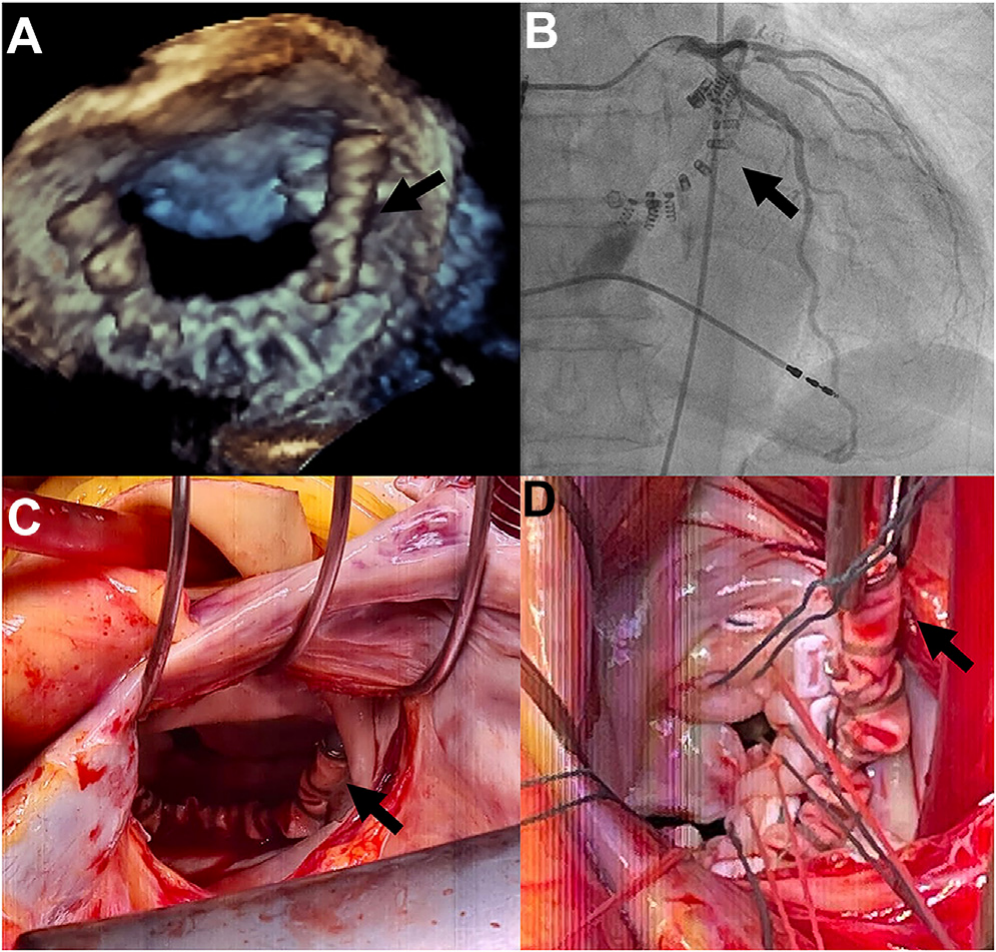

A 77-year-old woman presented with progressive dyspnea (New York Heart Association class III-IV) and pleural effusion due to congestive heart failure with severe mitral valve regurgitation (MR). Three years earlier, she was successfully treated for functional MR with a Cardioband (Edwards Lifesciences), a transcatheter annuloplasty device. Echocardiography showed left ventricular ejection fraction of 30%; severe MR with 2 jets, 1 centrally due to posterior leaflet tethering and 1 at the level of the annulus (Figure 1A; the arrow indicates the Cardioband); and chronic severe aortic valve insufficiency following structural valve deterioration with a central coaptation deficit. After multidisciplinary heart team evaluation and preoperative workup, including coronary angiography (Figure 1 B; the arrow indicates the Cardioband), the patient was scheduled for high-risk surgery.

Access to the mitral valve was established through a direct left atrial approach (Figures 1 C, D; the arrows indicate the Cardioband). Reconstruction was not feasible after the Cardioband implantation, causing a restrictive posterior leaflet with a perforation at P3, most likely damaged by screw anchors and a myxomatous anterior leaflet. To gain the maximal possible size for the prosthetic valve, the Cardioband had to be removed. Because of adhesions, complete removal of the screws was not possible without risk of damage to the fragile annulus. Therefore, the heads of the screws were cut and covered with endocardial plicatures, followed by implantation of a Hancock II 27-mm bioprosthesis (Medtronic). In addition, the patient underwent an aortic valve replacement (Epic Plus Supra aortic 23 mm; Abbott). The patient was discharged after an uneventful postoperative course. Follow-up examinations showed a moderately reduced left ventricular ejection fraction and competent valve functions with a sustainable improvement of heart failure symptoms (New York Heart Association class I).

This case exemplifies the emerging field of cardiac surgery: reoperation after transcatheter device implantation.

